# A human tissue-based assay identifies a novel carrion blowfly strain for maggot debridement therapy

**DOI:** 10.1038/s41598-022-16253-9

**Published:** 2022-07-16

**Authors:** Takuma Yoshida, Hiroka Aonuma, Saori Otsuka, Hidetoshi Ichimura, Erisha Saiki, Kosei Hashimoto, Manabu Ote, Sari Matsumoto, Kimiharu Iwadate, Takeshi Miyawaki, Hirotaka Kanuka

**Affiliations:** 1 Department of Tropical Medicine, The Jikei University School of Medicine, Tokyo, Japan; 2 Center for Medical Entomology, The Jikei University School of Medicine, Tokyo, Japan; 3 Department of Plastic and Reconstructive Surgery, The Jikei University School of Medicine, Tokyo, Japan; 4 Laboratory Animal Facilities, The Jikei University School of Medicine, Tokyo, Japan; 5 Department of Forensic Medicine, The Jikei University School of Medicine, Tokyo, Japan

**Keywords:** Translational research, Biological therapy, Entomology

## Abstract

Maggot debridement therapy (MDT) is a form of therapeutic wound treatment in which live fly larvae are used intentionally to debride necrotic tissues. MDT has been widely used to treat chronic wounds in humans or animals, such as diabetic foot ulcers. Larvae of a carrion blowfly, *Lucilia sericata* (green bottle fly), debride wounds by consuming necrotic tissue and removing pathogenic bacteria, promoting effective wound healing. Most medical *L. sericata* strains were initially collected from natural environments using animal meat as bait and reared on artificial protein-rich media or ground meat. It remains to be examined which strain would be more appropriate for MDT, whereas any method for evaluating the fly’s therapeutic potential in humans has not been available. A feeding assay was developed using minced human tissues obtained from surgical waste. To establish *L. sericata* strains highly eligible for MDT, carrion fly larvae were collected from 45 corpses subjected to forensic autopsy (such as decomposed bodies). Four corpse-derived *L. sericata* strains were obtained and evaluated using the feeding assay. One strain showed that its feeding activity was 1.4 times higher than the control strain used in conventional MDT. The body length of the adult fly of the corpse-derived strain was longer than the control, which was consistent with the observation that its cell size was enlarged. The human tissue-based assay developed in this study accurately evaluated the ability of fly larvae to debride necrotic wounds*.* The *L. sericata* strain newly established from human corpses harboring high feeding activity may offer a clinically significant improvement in MDT.

## Introduction

Diabetes is a major public health problem globally. From 1980 to 2014, the number of patients with diabetes increased from 108 to 422 million^1^. Because of recent lifestyle changes, diabetes is also increasing in prevalence in low- and middle-income countries more rapidly than in high-income countries^2,3^. Foot ulcers are common as neuropathic and vascular complications of diabetes. Ulcers form when full-thickness skin loss arises, and deeper structures such as muscle fascia and bone are exposed. These lesions often become chronic and get colonized by pathogenic bacteria, leading to limb amputation. A previous study indicated that a lower limb is amputated due to diabetes every thirty seconds^4^. Limb amputation following diabetic foot ulceration has a devastating effect on overall patient health and psychology^5,6^ and often leads to excessive premature death^7,8^; 5-year mortality for diabetic patients with foot ulcers tends to be approximately 40%^9^ and ranges from 53 to 90% in those with an ulcer of a subsequently amputated limb^10^. Thus, limb salvage is crucial and can preserve quality of life, minimizing the risk of condition decline and death.

Maggot debridement therapy (MDT), a treatment for chronic ulcers using fly larvae, has been applied for wound healing since ancient times. The French surgeon Paré described the stages of healing of a large wound in the skull in the presence of fly larvae in 1678^11^. MDT usually employs two closely related fly species larvae, the green bottle blowfly *Lucilia sericata* (Meigen) (Diptera: Calliphoridae) and the sheep blowfly *Lucilia cuprina* (Wiedemann) (Diptera: Calliphoridae)^12^. The larvae applied to wounds do not ingest any healthy tissues, and instead they exert debridement by consuming infected tissues, making the wound bed clean and healthy. In addition, the larvae improve the wound environment through their extracorporeal excretions and secretions (ES), which contain a variety of natural compounds with recognized activity against gram (+) and (−) bacteria^13^. Given the emergence of antibiotic-resistant bacteria, the disinfecting ability of the fly larvae is a vital alternative to antibiotics. Indeed, MDT effectively treats wounds infected with methicillin‐resistant *Staphylococcus aureus*^14^. MDT also promotes wound healing through granulation tissue formation^15^, which may be achieved through the ES by stimulating the proliferation of fibroblasts^16^, increasing angiogenesis^17^, and enhancing fibroblast migration^18^.

Although several fly species have been adopted for MDT so far^19–21^, *L. sericata* is the most commonly used for MDT based on numerous studies demonstrating its efficacy^22^. *L. sericata* larvae for MDT is approved by the Food and Drug Administration (FDA) as a prescription‐only medical device^23^ in the United States and regulated as drugs in some other countries. MDT is widely recognized as safe, efficacious, and inexpensive for wound debridement and limb salvage^24^. However, despite accumulated clinical evidence, MDT is less common in most countries and not covered by health insurance systems^25^. In general, health care providers and patients make decisions based on each therapy’s quality, efficacy, availability, cost, and practicality. Although its therapeutic potential is evident, MDT needs improvements to be recognized as an effective therapy for wound healing. One of the past advancements of MDT was the development of convenient mesh dressings to confine larvae in the wound^26^. On the other hand, due to the lack of screening to select better maggots, no significant advance has been made in the MDT regarding larvae functions, such as treatment efficacy, debridement, disinfection, and granulation. Thus, much room remains for ameliorating the fly strain itself by conferring a higher ability with these functions.

Quality assessment of larval activity has been performed using various assays with meat such as pork and beef instead of human tissue. A certain number of freely crawling larvae are placed and incubated on a mass of devitalized, non-sterile animal tissue (mainly muscle part). A slice of meat^27,28^ or a mass of minced meat^29^ is often used. These assays are intended to monitor insect growth and the reduction in substrate mass. It should be noted that these meats appear similar but are, in fact, quite different from the tissue of human ulcers, which mainly consists of necrotic skin, fat, muscle, and slough^30^. However, it has not been common to manage human tissue for this purpose because human necrotic tissues are generally not accessible.

This study aims to establish new blowfly strains potentially more suitable for MDT, and a human tissue-based assay using surgical waste was developed to analyze their features. One of larvae's expected features that may improve MDT is ingesting a larger amount of tissue than larvae of the standard medical strains. Candidate fly strains with a higher performance regarding debridement of human tissues were identified, and the human tissue-based feeding assay was applied to estimate how much tissue is ingested by larvae of those strains.

Carrion fly species, including *L. sericata*, lay their eggs in the decaying flesh of dead animals. Hatched larvae start scavenging the carrion by feeding so that the majority of the larvae in the wild are usually found in specific materials such as animal carcasses, which also include humans. To identify fly strains that show a strong feeding preference for human tissues, human corpses examined in forensic medicine, upon which fly larvae are found occasionally, were employed as a resource for collecting and screening wild *L. sericata*. Several *L. sericata* strains were successfully identified from human corpses during forensic autopsy.

The feeding ability of corpse-derived *L. sericata* strains was evaluated using minced human tissues. A strain that exhibited a higher feeding ability showed cell and body size enlargements compared with the reference strain. One of the main objectives of MDT is to remove necrotic, devitalized tissue that can delay wound healing. Necrotic skin tissues in wounds eventually become black, hard, and leathery due to a lack of blood flow. The corpse-derived *L. sericata* strains also well ingest necrotic tissues in addition to fresh ones when human necrotic tissue was provided to the larvae. The approach adopting a human tissue-based assay in this study may be helpful to identify new fly species/strains possibly applicable for MDT.

## Materials and methods

### Ethics statement

This study was performed in accordance with the Declaration of Helsinki. The Ethics Committee of The Jikei University School of Medicine approved the study protocol (permission number 27-052 [7936]). No patients were involved in the design or conception of this study.

### Human tissue

Human tissues were collected as medical waste from approximately sixty free-flap surgeries. The surgeries occurred between August 2015 and May 2017 at The Jikei University Hospital, Tokyo, Japan. The Ethics Committee of The Jikei University School of Medicine determined that informed consent was not required because this study used human tissues obtained from medical waste, and personal information was firmly anonymized by mixing those tissues during the mincing process. Information about this study was published on a bulletin board at The Jikei University Hospital. Normal tissues were sorted according to the organ such as skin, muscle, fat, and bone and stored at − 80 °C. Frozen tissues were defrosted once, minced using an electric meat grinder SG-50 (Fukunou Sangyo Co., Ltd., Hyogo, Japan) three times, and stored at − 80 °C until the feeding assays. Necrotic tissues were also collected from three free flap reoperations. These necrotic tissues, including skin, muscle, and fat, were pooled, stored at − 80 °C, and minced as described above. Due to the condition of tissue collecting, the tissues used in this study were not wholly sterile, and certain bacteria are assumed to be present.

### Porcine and bovine tissue

Pork-beef mince (a mixture of ground beef and pork meat) and pork fat mince were used in this study. Pork mince, beef mince, and pork fat tissue were purchased from Hanamasa Co., Ltd. (Tokyo, Japan). To prepare pork-beef mince, pork and beef minces were mixed at a one-to-one ratio and stored at − 20 °C. Pork fat tissue was minced using an electric meat grinder SG-50 (Fukunou Sangyo Co., Ltd., Hyogo, Japan) three times and stored at − 20 °C.

### Fly strain

A *L. sericata* strain available in Japan for medical use was obtained from Japan Maggot Company Ltd. (Okayama, Japan) and used as a control strain in this study. In order to establish new fly strains for MDT, dipteran larvae were collected from human corpses, which were subject to forensic autopsy in the western Tokyo area (Tama), Japan, from 2015 to 2016 (Table 1). Each larva was fed with pork-beef mince, separated into a plastic tube after pupation, and grown to adulthood.

**Table 1:** Specimens of fly larvae collected from forensic corpses in Western Tokyo (Tama area), Japan, from 2015 to 2016.

Subject no	Fly species	Site (city)	Post mortem interval (day)	Year
1	*Chrysomya pinguis, Lucilia sericata*	Chofu	14–21	2015
2	Not eclosed	Akishima	–	2015
3	*Liopygia crassipalpis*	Hachioji	–	2015
4	*Parasarcophaga similis*	Hino	20	2015
5	Phoridae gen. sp.	Machida	14	2015
6	*Chrysomya pinguis*	Hachioji	180	2015
7	*Lucilia sericata*	Machida	10	2015
8	*Lucilia sericata*	Tama	30	2015
9	*Boettcherisca peregrina*	Akishima	14	2015
10	*Lucilia sericata*	Fuchu	30	2015
11	Not eclosed	Hachioji	30	2015
12	Not eclosed	Nishitama	30	2015
13	*Chrysomya pinguis*	Nishitama	30	2015
14	*Chrysomya pinguis*	Nishitama	20	2015
15	Not eclosed	Akiruno	14	2015
16	Not eclosed	Hino	14–21	2015
17	Not eclosed	Chofu	–	2016
18	Not eclosed	Hachioji	–	2016
19	Not eclosed	Hino	7	2016
20	Not eclosed	Akishima	–	2016
21	Not eclosed	Nishitama	11	2016
22	*Chrysomya pinguis*	Nishitama	30	2016
23	Not eclosed	Nishitama	7	2016
24	*Lucilia sericata*	Hachioji	14	2016
25	*Lucilia sericata*	Hino	14	2016
26	*Lucilia sericata*	Ome	60	2016
27	Not eclosed	Fuchu	5–7	2016
28	*Lucilia sericata*	Hachioji	60	2016
29	*Boettcherisca peregrina*	Chofu	4	2016
30	*Lucilia sericata*	Hachioji	5	2016
31	Not eclosed	Akiruno	30	2016
32	Not eclosed	Machida	10	2016
33	Not eclosed	Fuchu	7	2016
34	*Lucilia sericata*	Machida	21	2016
35	*Lucilia sericata*	Fuchu	30	2016
36	*Lucilia sericata*	Machida	30	2016
37	Not eclosed	Hachioji	6	2016
38	Not eclosed	Nishitama	3	2016
39	Not eclosed	Akishima	30	2016
40	Not eclosed	Nishitama	3	2016
41	*Lucilia sericata*	Fussa	10	2016
42	*Lucilia sericata, Chrysomya pinguis*	Fussa	30–60	2016
43	Not eclosed	Hino	60	2016
44	Not eclosed	Machida	60	2016
45	Not eclosed	Ome	30–60	2016

### Fly rearing

About forty adult flies in a cage (bottom 27 cm × 27 cm, top 25 cm × 25 cm, height 27 cm) were allowed to lay eggs in 10 g of fresh pork liver. Next, eggs were transferred to 20 g of pork-beef mince (50% pork, 50% beef) placed in a plastic container (101 mm in diameter, 45 mm in depth). Larvae that hatched from the eggs were kept at 27 °C for 5 days. Approximately 100 individuals of wandering third instar larvae were then collected into a paper cup containing shredded paper, transferred into a new cage, and kept for 7 days to pupariate. Adult flies were fed with a piece of medical cotton wool (Hakujyuji Co., Ltd, No.5, 5 cm × 5 cm) soaked in distilled water and 4 g of mixed fly diet (50% protein powder [SAVAS Shape & Beauty, 4902777308531, Meiji Co., Ltd., Tokyo, Japan] and 50% sugar) placed in the cage. The cage was kept at 27 °C in 12 h light and 12 h darkness cycles. The water-soaked cotton and the mixed fly diet were replaced every 3–4 days.

### Species identification of fly

Fly morphological traits were used for estimating fly species. Images of the whole body of each adult were taken using a stereomicroscope S6E (Leica Microsystems GmbH, Wetzlar, Germany) equipped with an ILCE-5100 camera (Sony Corporation Ltd., Tokyo, Japan) with a NY-1S adaptor (Micronet Inc., Saitama, Japan). Morphological characteristics (number of para-vertical setulae and occipital bristles, color of basicosta, and number of setae on the scutum of the mesonotum) were used to distinguish each fly image as reported previously^31,32^.

Fly species identified as *L. sericata* or *L. cuprina* by morphological traits were then confirmed by sequencing mitochondrial and nuclear genes as follows. The whole body of each adult fly was used for DNA purification after obtaining its offspring. DNA was extracted using a conventional method: each fly was homogenized in 200 µl of Buffer A (0.1 M Tris [pH 9.0], 0.1 M EDTA, 1% SDS, and 0.5% DEPC), and the homogenized mixture was incubated at 70 °C for 30 min. After adding 44.8 µl of 5 M potassium acetate, the mixture was incubated on ice for 30 min. The supernatant was collected by centrifugation at 20,400×*g* at 4 °C for 15 min and mixed with 90 µl of isopropanol. Precipitated DNA was collected after centrifugation at 20,400×*g* at 4 °C for 20 min, rinsed with 70% ethanol, and dried. DNA was diluted in TE and prepared for sequencing. Three genes were chosen for sequencing: mitochondrial cytochrome oxidase subunit I (*COI*), *28S* rRNA (*28S*), and *period* (*per*). The primers 5′-CTG CTA CTT TAT GAG CTT TAG G-3′ and 5′-CAT TTC AAG YTG TGT AAG CAT C-3′ reported previously^33^ were used to amplify a region of approximately 350 bp of the *COI* gene. The primer pairs, 5′-CCC CCT GAA TTT AAG CAT AT-3′ and 5′-GTT AGA CTC CTT GGT CCG TG-3′, and 5′-GCC TTC AGA TAC GGT CAA AC-3′ and 5′-CCG AGT GTG GTT TGG AGA TT-3, as described previously^34^, were used to amplify each region of approximately 780 bp of the *28S* gene and 790 bp of the *per* gene, respectively. Polymerase chain reaction (PCR) was performed to amplify these target regions in accordance with the procedure reported together with primers except using TaKaRa Ex Taq (Takara Bio Inc., Japan). PCR products were confirmed by gel electrophoresis and purified using a QIAEX II Gel Extraction Kit (QIAGEN). PCR products were then subjected to sequence analysis (Fasmac Co., Ltd., Japan) using the same primers. Sequence data was analyzed for similarity using NCBI Nucleotide BLAST.

### Feeding assay

For the feeding assay, first instar larvae hatched from the eggs were used. Each diet (16–40 g; pork-beef mince, pork fat mince, human fat, skin, muscle mince, and human necrotic tissue mince) was mixed with half distilled water and served to 30–100 first instar larvae. These larvae were kept at 27 °C in the cage and developed into adults. The number of surviving individuals (larvae, pupae, and adults) was counted to calculate the survival rate.

The weight of each individual and its developmental stage (larvae, pupae, and adults) were evaluated. The weight of each individual was measured using an electronic microbalance MSA6.6S-000-DM (Sartorius AG, Göttingen, Germany) with an accuracy of 0.001 mg. The dry weight of larva was measured following lyophilization of larvae using a freezing vacuum drying machine FreeZone 4.5 (Labconco Co., Kansas City, MO, USA).

The stage of each larva was determined using the morphological feature and behavior of the larvae. Images of the posterior spiracular slits of each larva were captured using a stereomicroscope equipped with a camera as described above. A larva carrying three separated spiracular slits and a complete peritreme with an inter slit projection was judged as a third instar larva (early and late). Of these larvae, a larva that stopped feeding and wandered on dry areas rather than wet ones was considered as a late stage of the third instar.

The amount of diet ingested by larvae was determined as follows. First, 6 g of diet was served to 10 first instar larvae, and the diet remaining at 5 days after hatching was measured using an electronic microbalance. Then, the net amount of the ingested diet was divided by the number of larvae to calculate the food consumption per larva.

### Body size, wing vein length, and trichome density

Body size, vein length, and trichome density in the wings were compared among fly strains. Thirty first instar larvae were fed on 18 g of pork-beef mince. The pupae and adults were collected at 11 days and 26 days after hatching, respectively. Images of the pupal and adult body were captured using a stereomicroscope equipped with a camera as described above. Pupal and adult body lengths from the vertex of the head to the posterior tip of the abdomen were measured. Adult wings were washed three times with a solution containing 70% ethanol and 0.3% Triton X and mounted in 80% glycerol. Images of adult wings were captured using a Leica DM2500 microscope equipped with Leica DFC300 FX digital camera (Leica Microsystems GmbH, Wetzlar, Germany). The dm-cu length of the wing vein of female adults was measured. Trichome density in a 150-pixel square was calculated using the freeware Fijiwings (https://sourceforge.net/projects/fijiwings/).

### Immunohistochemistry

The cell size of the larval fat body was examined as follows. Ten larvae at 4 days after hatching were collected on an iced petri dish and washed with ice-cold phosphate-buffered saline (PBS). The larvae were dissected in PBS and then fixed in 4% paraformaldehyde for 5 min on ice, then incubated for 60 min at room temperature. Fixed whole tissues were washed with PBT (PBS + 0.1% Triton X-100) three times for 20 min each and incubated with PBTn (PBT + 5% goat serum) for 30 min at room temperature. The tissue was washed with PBT 4 times for 10 min each and incubated with Alexa Fluor 555 Phalloidin (Thermo Fisher Scientific, MA, USA) containing 1% BSA for 2 h at room temperature. The tissue was washed with PBT 4 times for 10 min each and incubated with TO-PRO-3 iodide (Thermo Fisher Scientific, MA, USA) for 15 min. The tissue was washed with PBT 4 times for 10 min each and kept in PBS at 4 °C. The fat body was collected from the tissue and mounted using Vectashield (Vector Laboratories, CA, USA). Images were acquired on a confocal microscope TCS-SP8 (Leica Microsystems GmbH, Wetzlar, Germany). Ten fat body cells per individual larva were used to measure cell size using NIH Image-J (https://imagej.nih.gov/ij/).

### Larval excretions and secretions (ES)

Fly eggs were washed with ultrapure water, 1% sodium hypochlorite solution, 70% ethanol, and ultrapure water. The washed eggs were placed in a plastic tube. One hundred first instar larvae that hatched from eggs were incubated in 200 μl of PBS for 1 h at room temperature. The supernatant containing larval ES was collected in a 1.5 ml tube. The larval ES solution was concentrated using a centrifugal filter Amicon Ultra-0.5 (UFC500324, Merck Millipore, Darmstadt, Germany). The protein concentration of the larval ES solution was determined using a BCA Protein Assay Kit (23225, Thermo Fisher Scientific, MA, USA) and a spectrometer GeneQuant *pro* (GE Healthcare, IL, US) in accordance with the manufacturers’ protocols. The larval ES solution was aliquoted and stored at − 30 °C until use.

### Cell proliferation assay

Human foreskin fibroblasts (HFF) cells were cultured in Dulbecco’s modified Eagle’s medium (DMEM) supplemented with 10% fetal bovine serum (FBS) and 10 μg/ml gentamicin at 37 °C and 5% CO_2_. To examine cell proliferation, 0.6 × 10^4^ HFF cells per well in a 96-well plate were cultured for 24 h. Larval ES solution was diluted with PBS to adjust protein concentration and added to each well. After incubating these cells for 48 h at 37 °C, a Cell Counting kit-8 (CK-04, Dojindo, Kumamoto, Japan) was used to determine viable cell numbers in accordance with the manufacturer's instructions. The absorbance in each well was measured using a microphotometer EnSpire2300 (PerkinElmer, MA, USA).

### Fibrin dissolution assay

Ten milliliters of 5 mg/ml fibrinogen solution dissolved in 0.2 M sodium borate buffer (pH 7.8) (067-03693, Wako Pure Chemical Co., Tokyo, Japan) were added to a petri dish (100 mm in diameter). Five hundred microliters of 20 unit/ml human thrombin solution in a 0.2 M sodium borate buffer (33839-46, Nacalai Tesque Inc., Kyoto, Japan) was added to the dish, stirred immediately, incubated for 30 min to form a coagulated fibrin plate. Fifty microliters of each cell culture supernatant collected in the scratch assay (as described below) were added to the center of the plate. The plates were incubated for 40 h at 37 °C. Images of the fibrin-dissolved area were captured, and the dissolved area was measured using NIH Image-J.

### In vitro scratch assay

HFF cells (0.6 × 10^4^ per well) in a 24-well plate were cultured at 37 °C. After forming a confluent cell monolayer, the cells were scraped with the top of a 200 μl pipette tip in a straight line to create an artificial gap (scratch). The debris from the scratched cells was removed, and the edge of the scratch was smoothed by washing the cells twice with PBS. Five hundred microliters of DMEM containing 1% FBS and 10 μg/ml gentamicin were added to each well. Fifty µl of PBS or larval ES solutions diluted to adjust the protein concentration with PBS were supplemented into the medium. After 12 h, the cell culture supernatants were collected and kept at − 80 °C for the fibrin dissolution assay (as described above). Images of each well after 0 or 12 h of culture were captured, and the closure areas (cell-covered gap) were measured by comparing the two images for each well using NIH Image-J. The closure areas of the PBS-treated cells at 12 h were adjusted to have a mean value of 100. The closure areas of samples were calculated by dividing each value by that of PBS-treated one obtained in the same experiment.

### De novo assembly and analysis of RNA-seq data

Fly eggs were washed with ultrapure water, 1% sodium hypochlorite solution, 70% ethanol, and then ultrapure water again. Washed eggs were placed in a plastic tube. The first instar larvae that hatched from the eggs were collected for RNA-seq analysis. TRIzol (Invitrogen, CA, USA) was used to isolate the total RNA from these larvae. The total RNA samples were sequenced on an Illumina HiSeq 2500 (100 bp, paired-end) and analyzed by Hokkaido System Science Co. Ltd. (Hokkaido, Japan). Sequenced reads were trimmed using Cutadapt^35^ and Trimmomatic^36^. Because any reference genome or transcriptome datasets of *L. sericata* were not available at the beginning of this study, de novo transcriptome assembly of RNA-seq data was performed using Trinity^37^. Gene expression levels among fly strains were estimated with RSEM^38^ and compared using edgeR^39^. Assembled transcripts were manually annotated, referring to available genetic information of other brachyceran fly species.

### Statistical analysis

Student’s *t*-test and two-sided log-rank test were performed in this study using *R* version 3.4.1 (http://www.r-project.org). Probability values less than 0.05 were considered statistically significant.

## Results

### Development of a human tissue-based feeding assay for fly larvae

**Figure 1:**
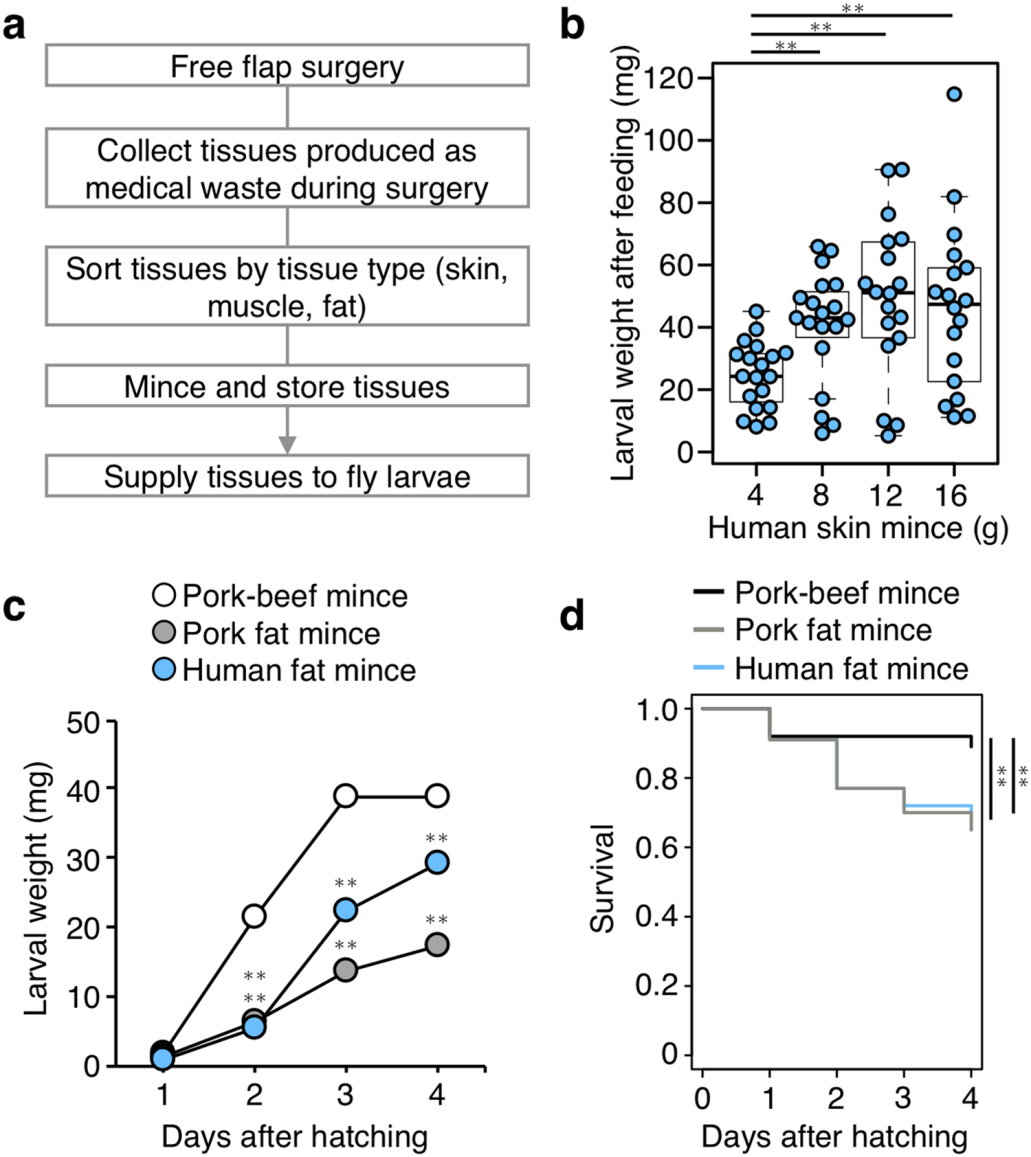
Development of a human tissue-based feeding assay for *L. sericata* larvae. (**a**) A flowchart describing human tissue preparation for making the diet of fly larvae. During free flap surgery, human tissues produced as medical waste were collected, sorted by type of tissue, minced, and supplied to *L. sericata* larvae. (**b**) Weight of larvae at 4 days after hatching and fed with human skin mince diet. The indicated amounts of diet were supplied to each group (n = 20). Each dot represents the weight of an individual larva. Boxplots: center line, median; box range, 25th–75th percentiles; whiskers denote minimum–maximum values. Student’s *t*-test, **p < 0.01. (**c**,**d**) Weight and survival ratio of larvae fed on 40 g of pork-beef mince, pork fat mince, or human fat mince. n = 100 per group. The mean values are shown ± SEM in (**c**). Student’s *t*-test (**c**), two-sided log-rank test (**d**). **p < 0.01. See also Supplementary Data 1 for data of experiment, exact sample sizes, and *p* values.

The feeding and growth characteristics of fly larvae when fed on human tissues were examined at first. Human tissues prepared as medical waste from surgery were sorted according to organ type, minced, and supplied as a diet for larval feeding (Fig. 1a). A different amount of human skin mince was supplied to fly larvae collected immediately after hatching (20 individuals each). To evaluate larval growth, larval weight was measured 4 days after hatching, when most of the flies became wandering third instar larvae. As a result, any human skin mince used in this experiment was able to support larval growth, increasing their weight (Fig. 1b). Larvae fed with 12–16 g of human skin mince did not show any additional increase in weight compared with larvae fed with 8 g (8 g vs. 12 g: *p* = 0.22, 12 g vs. 16 g: *p* = 0.69, Fig. 1b), suggesting that feeding with 0.4 g of human skin mince is enough for one larva to grow fully.

Next, the effect of feeding human and pork tissues (fat mince) to fly larvae was compared on their growth and survivability. Pork-beef meat mince was used as a standard diet. The weight of larva fed with human fat was significantly higher than that of larva fed with pork fat (*p* < 0.01 at 4 days after hatching, Fig. 1c). The survival rate of larvae fed with human fat was equivalent to that of larva fed with pork fat (*p* = 0.45, Fig. 1d). The pork-beef mince showed higher values of both growth and survival than human or pork fat mince (Fig. 1c,d). These results suggested that the nutritional value of the human tissue is comparable to or higher than pork tissue.

### Human tissue-dependent growth and survivability of fly larvae

**Figure 2:**
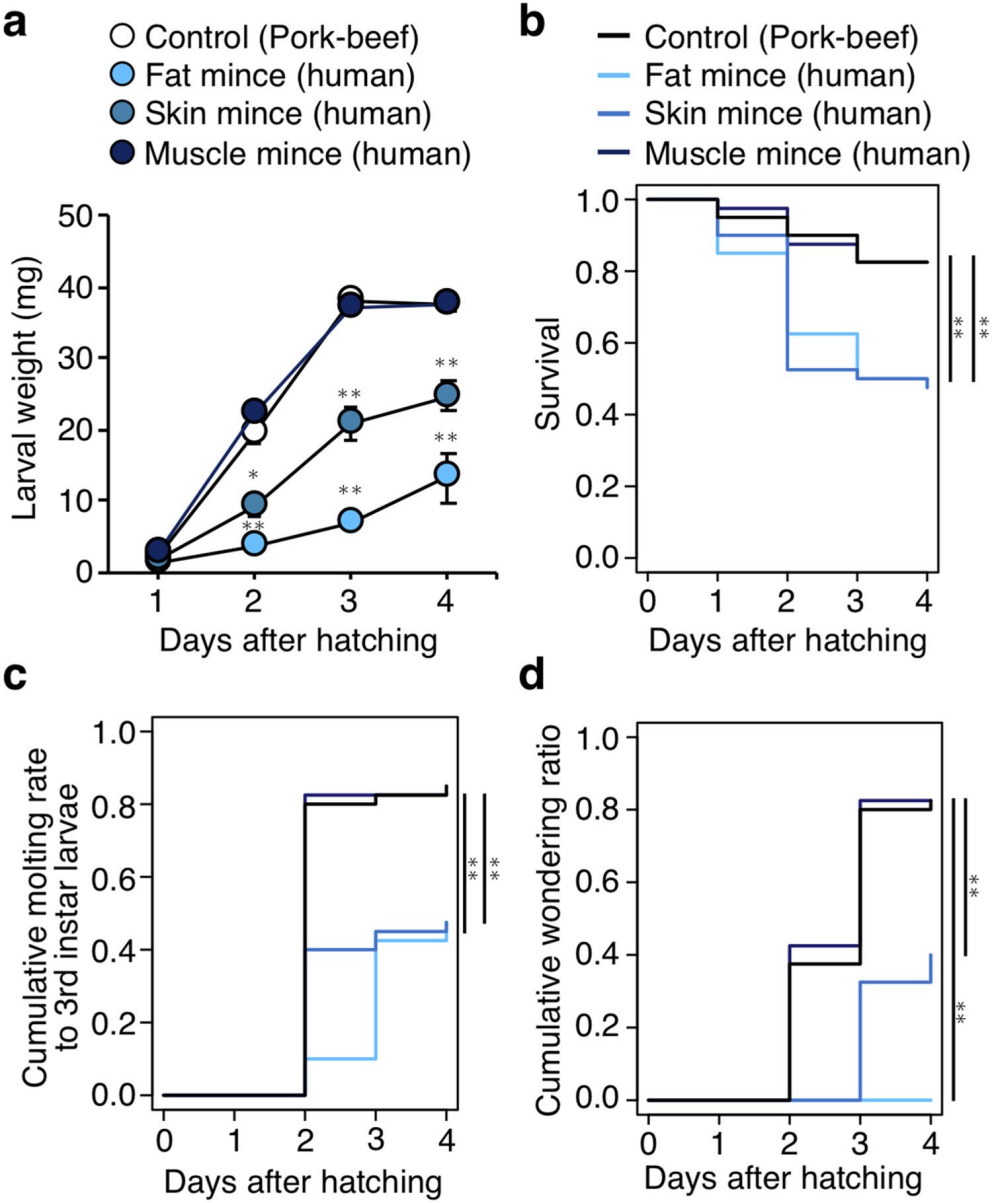
Human tissue-dependent growth and survivability of *L. sericata* larvae. (**a**,**b**) Weight and survival ratio of larvae fed on 16 g of pork-beef mince, human fat mince, human skin mince, or human muscle mince. n = 40 per group. The mean values are shown ± SEM in (**a**). Student’s *t*-test (**a**), two-sided log-rank test (**b**), *p < 0.05, **p < 0.01. (**c**,**d**) Developmental rates of larvae (third instar and wandering) fed on 16 g of pork-beef mince, human fat mince, human skin mince, or human muscle mince. n = 40 per group. Two-sided log-rank test, **p < 0.01. See also Supplementary Data 1 for data of experiment, exact sample sizes, and *p* values.

To examine which human tissues are appropriate for feeding flies, larvae were fed with different minces prepared from human fat, skin, and muscle. Pork-beef meat mince was used as a standard diet. Larval weights at 4 days after hatching varied depending on which tissue the larvae ingested; human muscle mince supported larval growth to a similar level as the pork-beef meat mince, whereas human fat mince showed a relatively low ability to support larval growth (Fig. 2a). The survival ratio of larvae fed on human muscle mince was significantly higher than that for other human tissues (*p* < 0.01, Fig. 2b). It was also observed that the transition of larval developmental stages was significantly affected by the tissue that larvae ingested. Larvae fed with human muscle developed into third instar larva as early as ones fed on pork-beef meat mince (Fig. 2c,d). On the other hand, although most of the larvae fed on human skin or muscle already started wandering at 4 days after hatching, larvae fed on human fat showed a significant delay in development (*p* < 0.01, Fig. 2c). No wandering larvae (late third instar) raised on human fat were observed (Fig. 2d), suggesting that human fat itself is insufficient to support proper larval development.

Next, the feasibility of a human tissue-based feeding assay was considered in terms of the availability of human tissues. The results indicated that human muscle was the most nutritious diet appropriate for larval growth and development, and the human skin was the next best (Fig. 2a–d). Given the practical difficulty in securing human muscle tissues as medical waste, the human skin, frequently obtained from flap surgeries, was used as a diet resource for the human tissue-based feeding assay in this study.

### Collecting carrion fly species from human corpses

**Figure 3:**
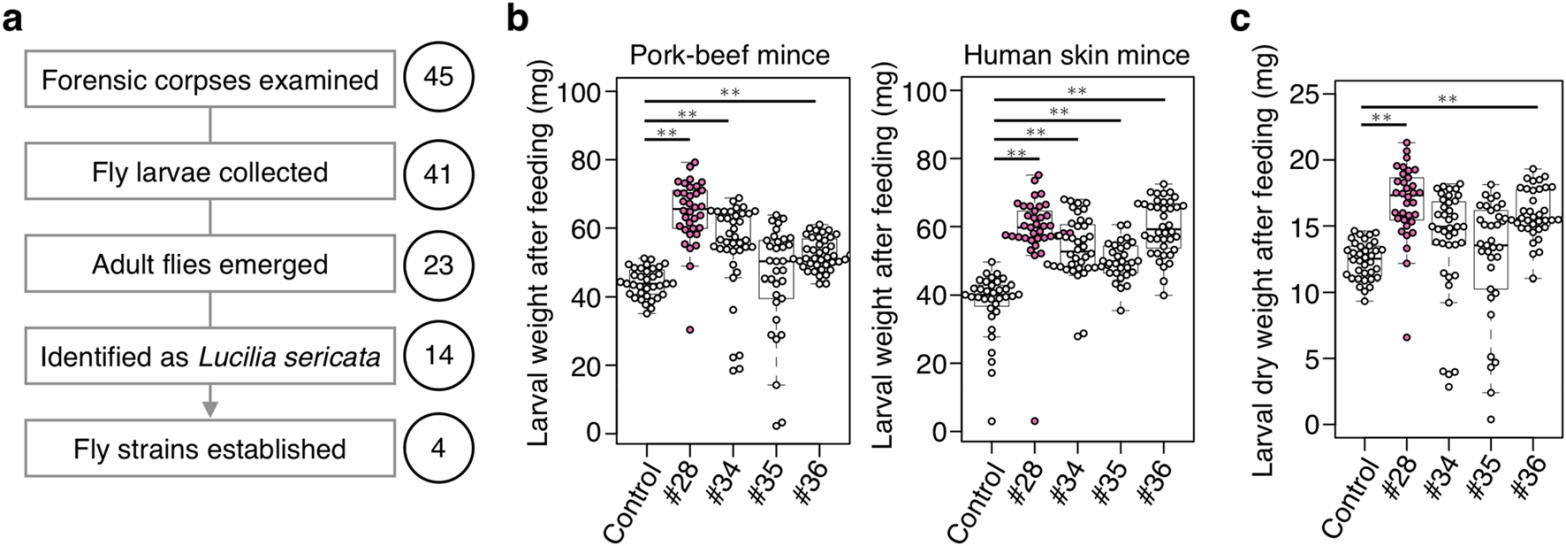
Analysis of carrion fly species collected from human corpses. (**a**) A flow chart of collecting fly larvae and identifying *L. sericata* from forensic corpses. Each number indicates the human corpse examined. Four *L. sericata* strains (#28, #34, #35, #36) were established. (**b**) Weight of larvae supplied with 24 g of pork-beef mince (left) or human skin mince (right). Weight was measured at 5 days (left) and 4 days (right) after hatching. n = 40 per group. (**c**) Dried weight of larvae at 5 days after hatching supplied with 24 g of pork-beef mince. n = 40 per group. In (**b**) and (**c**), each dot represents the weight of an individual larva. Boxplots: center line, median; box range, 25th–75th percentiles; whiskers denote minimum–maximum values. Student’s *t*-test, **p < 0.01. See also Supplementary Data 1 for data of experiment, exact sample sizes, and *p* values.

Fly larvae were collected from 45 forensic corpses (mostly being decomposed) found in Western Tokyo (Tama area), Japan, from 2015 to 2016 (Table 1). Fly adults emerged from larvae on 23 corpses, and characterization of the morphological traits identified adults from 14 corpses as *L. sericata* (Fig. 3a). Four strains (#28, #34, #35, #36) were established from independent corpses and subsequently confirmed as *L. sericata* by analyzing nucleotide sequences of the mitochondrial COI, nuclear 28S rRNA, and *period* genes.

Larvae of these corpse-bone strains were fed on human skin mince or pork-beef mince, followed by measuring the weight of each larva. Each larva of the four corpse-derived strains was 1.33–1.58 times heavier than the control strain after feeding on human skin mince (*p* < 0.01, Fig. 3b). A similar result was obtained from the experiment using the pork-beef mince (Fig. 3b). To avoid overestimating the water content in the larval body, the dry weight of each larva was measured. Significant differences were also observed among larvae of two corpse-derived strains (#28 and #36) compared with the control strain (*p* < 0.01, Fig. 3c). These results suggested that *L. sericata* strains sourced from the human corpses may be comparable to or better than standard medical strains in the consumption of human tissues.

### A corpse-derived fly strain that ingests human tissue effectively

**Figure 4:**
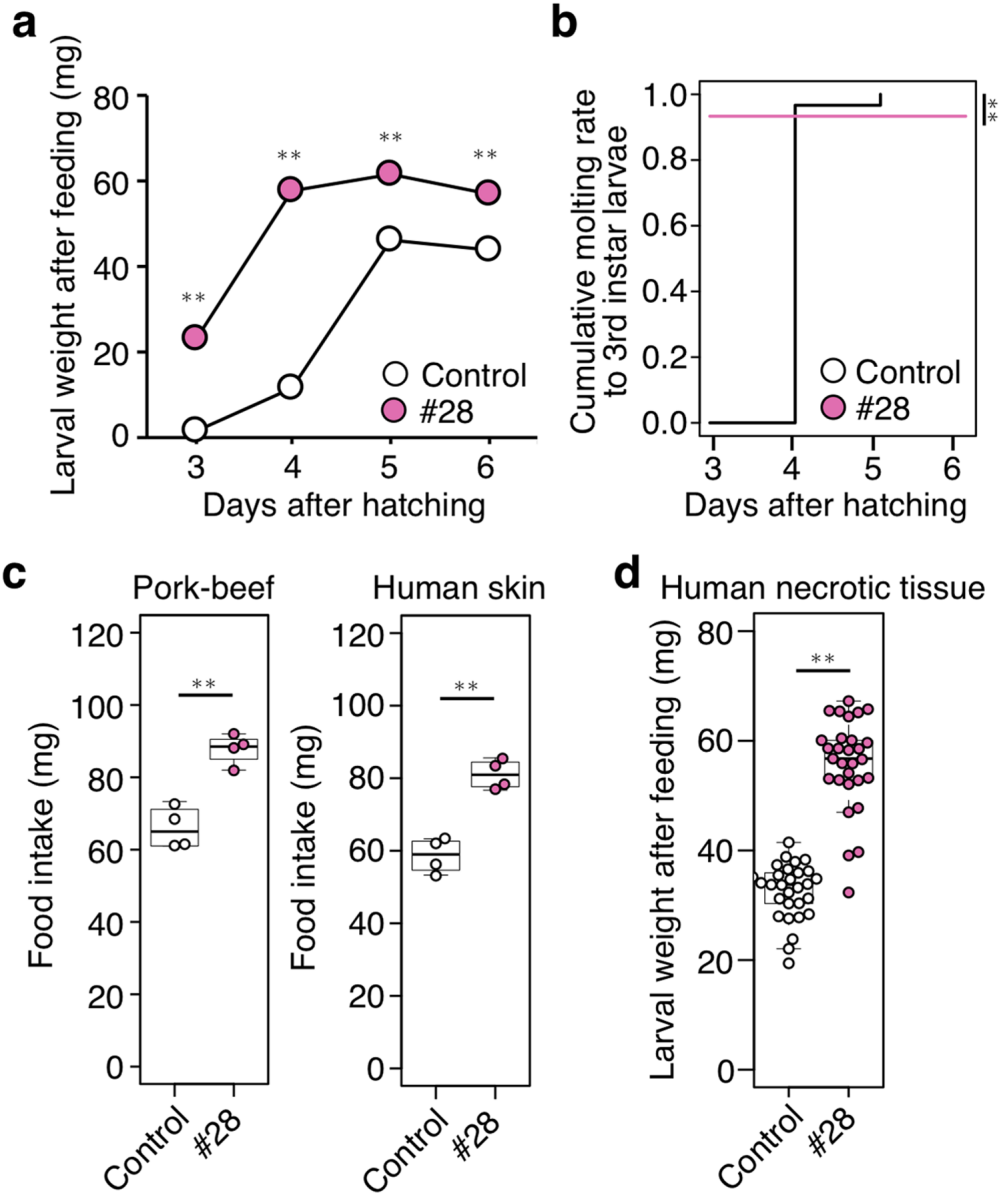
A corpse-derived fly strain that ingested human tissue effectively. (**a**,**b**) Weight (**a**) and developmental rate (third instar larvae) (**b**) of larvae supplied with 18 g of pork-beef mince. n = 40 per group. The mean values are shown ± SEM in (**a**). Student’s *t*-test (**a**), two-sided log-rank test (**b**). **p < 0.01. (**c**) Amount of food intake by larvae at 5 days after hatching supplied with 6 g of pork-beef mince (left) or human skin mince (right). n = 4 per group. (**d**) Weight of larvae at 5 days after hatching supplied with 18 g of human necrotic tissue mince. n = 30 per group. In (**c**) and (**d**), each dot represents the value of an individual larva. Boxplots: center line, median; box range, 25th–75th percentiles; whiskers denote minimum–maximum values. Student’s *t*-test, **p < 0.01. See also Supplementary Data 1 for data of experiment, exact sample sizes, and *p* values.

One of the corpse-derived strains (strain #28), which gained significantly more weight than the control when fed on human skin (Fig. 3b,c), was selected and subjected to further analysis. Strain #28 grew faster and molted into the third instar larva earlier than the control (Fig. 4a,b), indicating that these larvae may ingest a larger amount of human tissue. To evaluate the larval feeding ability more accurately, the food intake was quantified by measuring the remaining diet after feeding. The amount of pork-beef mince or human skin mince consumed by larvae of strain #28 was significantly larger than the control at 5 days after hatching (*p* < 0.01, Fig. 4c), confirming that food intake per unit time differed according to the fly strain. Strain #28 significantly grew heavier than the control when fed on necrotic tissue (p < 0.01, Fig. 4d), indicating its substantial feeding ability on necrotic tissues. These results suggested that corpse-derived strain #28 has a higher feeding ability on human tissues and can be of practical use for MDT.

### Correlation between feeding ability and body size of corpse-derived flies

**Figure 5:**
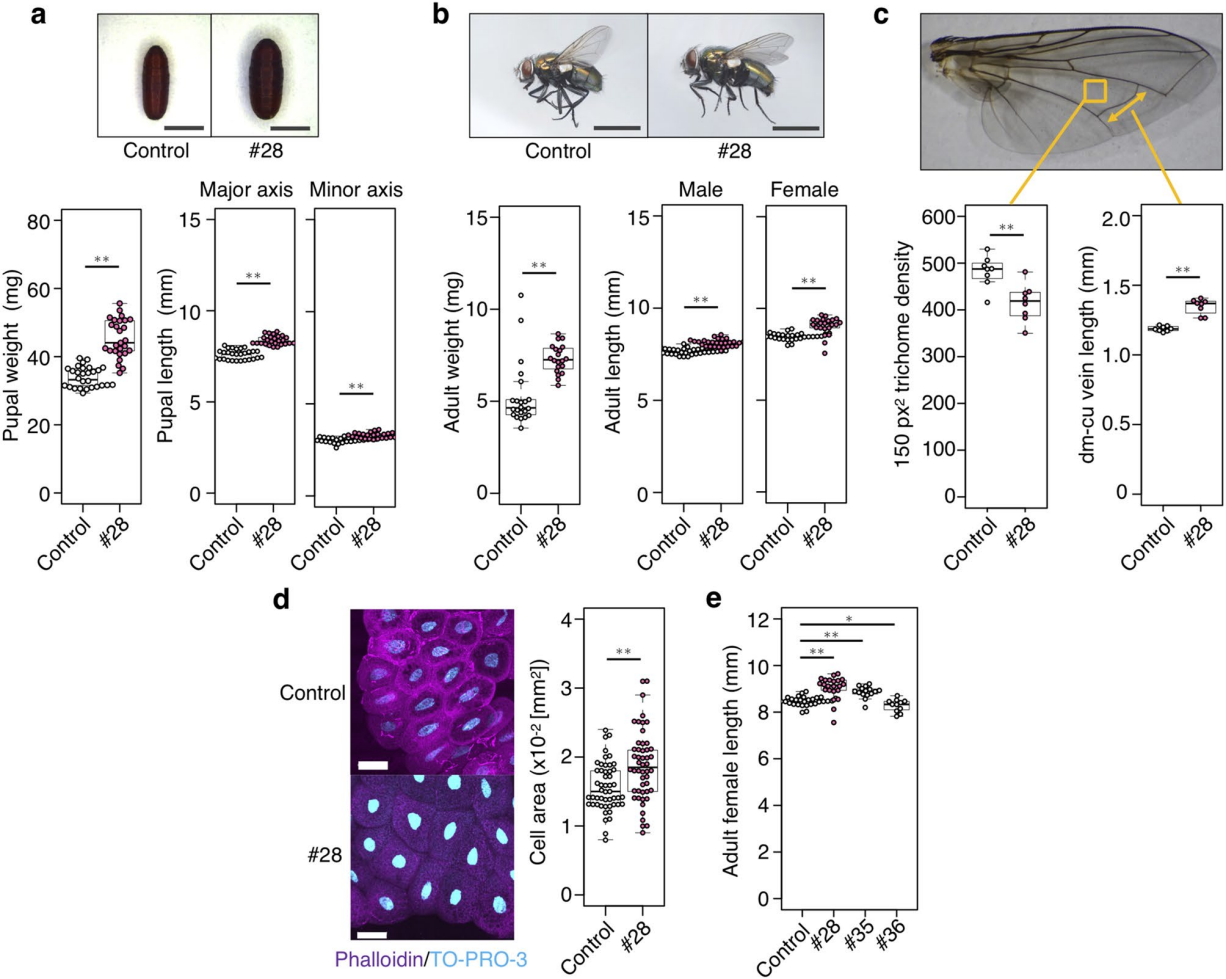
Correlation between feeding ability and body size of the corpse-derived flies. (**a**) Weight and length (major and minor axes) of pupa at 11 days after hatching. n = 30 per group. Bar = 4 mm. (**b**) Weight and length of adults at 26 days after hatching. n = 30 per group. Bar = 5 mm. (**c**) Trichome density and vein length of the wings of female adults at 26 days after hatching. n = 8 per group. (**d**) Size of cells in the fat body of larvae at 4 days after hatching. n = 40 per group. Magenta, phalloidin (actin); blue, TO-PRO-3 (nuclei). Bar = 100 µm. (**e**) Length of female adults of corpse-derived fly strains. Note that strain #34 was not included due to failure to maintain the strain. Each dot represents the value obtained from an individual specimen (**a**–**e**). Boxplots: center line, median; box range, 25th–75th percentiles; whiskers denote minimum–maximum values. Student’s *t*-test, *p < 0.05, **p < 0.01. See also Supplementary Data 1 for data of experiment, exact sample sizes, and *p* values.

Higher nutrition intake from ingested human tissues may correlate with various fly phenotypes such as physical enlargement, increasing basal metabolic rate, or precocious development. To clarify if the higher feeding ability of the corpse-derived strain related to other features besides faster development, body sizes among the strains were compared. Instead of larva, the body lengths of pupae and adults were measured because preservation may hinder accurate measurement of larval body length^40^. The body sizes of pupae and adults of strain #28 were significantly larger than the control, consistent with the results from measuring their weights (Fig. 5a,b).

Then, the cell size of strain #28 was analyzed to examine whether a difference in cell size or cell number contributed to the body size enlargement. Each epidermal cell of the fly wing has a single hair, called a trichome; therefore, the density of trichomes indicates cell size. The number of trichomes within a defined wing area of strain #28 was lower than that of the control (Fig. 5c). It was also observed that the length of a wing veins of strain #28 was longer than that of the control (Fig. 5c), suggesting the presence of larger cells in the body of strain #28. As expected, larger cells were also present in the fat body of strain #28 (Fig. 5d). In addition to strain #28, the other “big eater” strains also showed large adult body sizes (Fig. 5e). These results suggested that the body size of carrion flies can be a reliable marker for their potential for food consumption.

### Cell proliferation activity of larval excretions and secretions

**Figure 6:**
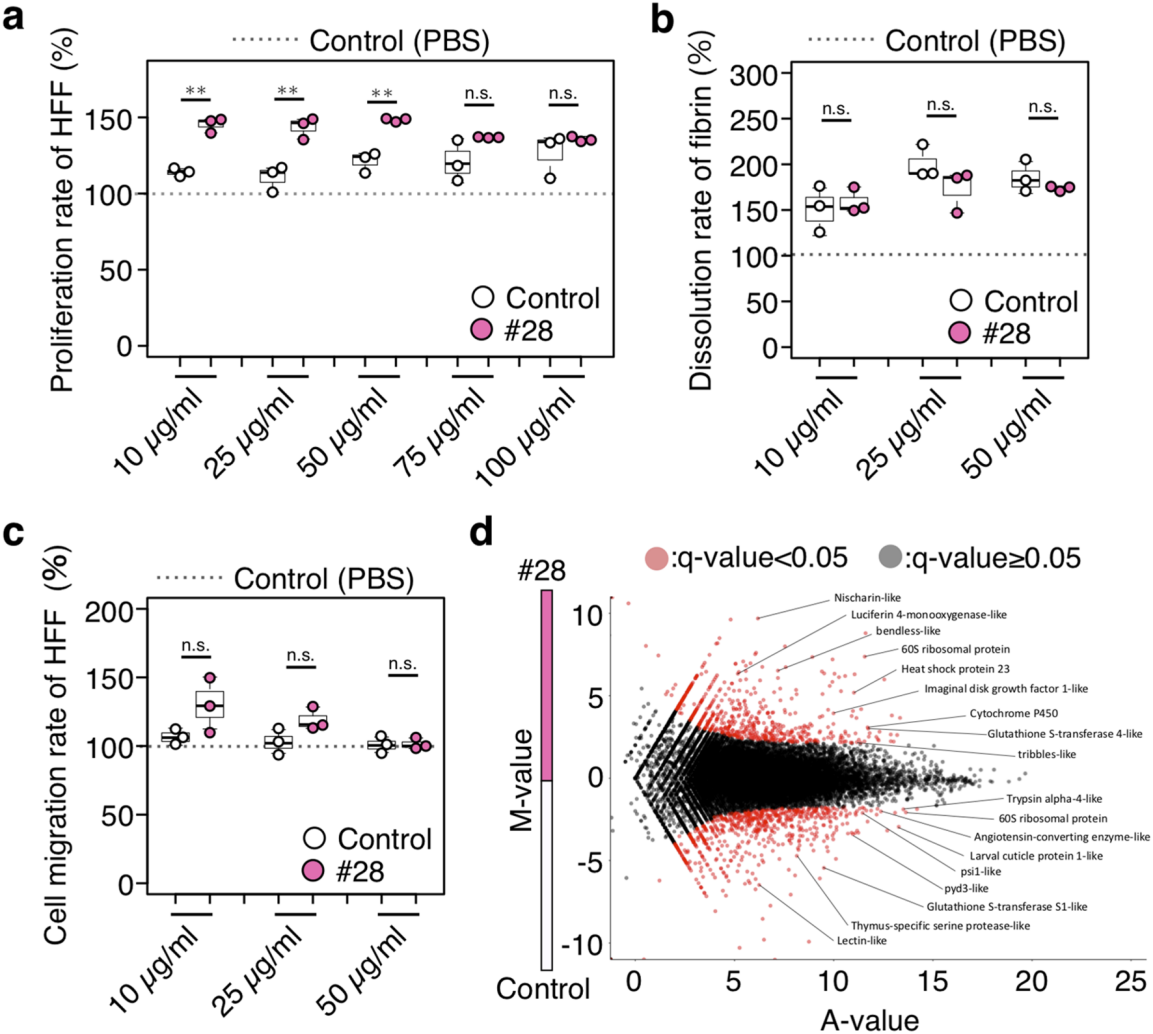
Cell proliferation activity of larval excretions and secretions (ES). (**a**) Proliferation rate of HFF cells treated with the indicated concentration of larval ES. n = 3 per group. (**b**) Dissolution rate of fibrin by culture supernatant of HFF cells treated with the indicated concentrations of larval ES. n = 3 per group. **c** Cell migration rate of HFF cells treated with the indicated concentration of larval ES. n = 3 per group. Each dot represents the value obtained from an individual specimen (**a**–**c**). Each rate is expressed as the percentage of larval ES-treated condition versus PBS only treatment (shown as 100%). Boxplots: center line, median; box range, 25th–75th percentiles; whiskers denote minimum–maximum values. Student’s *t*-test, **p < 0.01. n.s., not significant. See also Supplementary Data 1 for data of experiment, exact sample sizes, and *p* values. (**d**) MA plots of RNA sequence reads of control strain, compared with strain #28. Genes with q-value < 0.05 are labeled in red. M, log_2_ fold change, A, averaged log_2_ reads.

It has been assumed that larval ES are essential to enhance wound healing by stimulating human cell proliferation and migration^16,18^. To evaluate the capability of strain #28 in wound repair, the proliferation and migration rate of HFF cells treated with its larval ES were measured. The larval ES-stimulated cellular activity of fibrinolysis (dissolution of fibrin clots) was also examined. Each larval ES collected from strain #28 and the control was confirmed to promote the proliferation of HFF cells and dissolved fibrin clots (Fig. 6a,b). The proliferation rates of HFF cells incubated with the larval ES of strain #28 were higher than those of the control at concentrations of 10, 25, and 50 μg/ml (Fig. 6a). On the other hand, the fibrin dissolution activity of HFF cells induced by the larval ES of strain #28 was similar to the control larval ES (Fig. 6b). It was noted that no effect on cell migration rate was observed when the larval ES of strain #28 or the control was added to HFF cells (Fig. 6c).

Finally, to obtain molecular insight into the higher ability of strain #28, the de novo transcriptome assemblies of RNA-seq data in first instar larvae immediately after hatching were compared between strain #28 and the control. Thresholding genes with q values < 0.05 yielded 2,993 genes as differentially expressed genes (DEGs); 1,623 genes were upregulated, and 1,370 genes downregulated in strain #28 (Fig. 6d). A manual annotation predicted that the DEGs might contain heat-shock proteins, ribosomal proteins, glutathione S-transferases, cytochromes P450, cuticle associating factors, and insulin-like peptide signaling pathway associating factors. This result implied that the fly abilities such as debriding damaged tissue and enhancing granulation tissue formation may be achieved by the differentially regulated genes in each strain.

## Discussion

MDT has long been used for the treatment of necrotic wounds without refining fly species and strains due to the lack of available methods to evaluate the fly’s ability in debriding human tissues. In this study, adopting medical waste from surgery on human patients opened the way to measure how much larvae ingest human substrates for the first time. In addition to previous studies^41,42^, larval growth rates have been demonstrated to vary depending on the animal species and the type of tissues they intake, suggesting that applying human tissue instead of edible meat from livestock is needed for selecting fly strains toward improving MDT. In particular, a feeding assay using human skin, in which the elasticity and viscoelasticity are unique^43^, can be a highly representative model for MDT.

To the best of our knowledge, this is the first modern study employing human corpses as a resource for wild flies to establish new strains applicable for MDT. *L. sericata* is known for its beneficial uses in forensic entomology and is one of the primary insects from cadavers used as an indicator to estimate the postmortem interval^44^. Development time at a particular temperature varies among *L. sericata* populations depending on the local climate where the fly was obtained, suggesting the occurrence of adaptation of flies to their environment and possibly altering the fly’s phenotype^45^. Indeed, our study revealed that the feeding amount and growth rate of larvae, which is essential to determine the application interval for MDT, varies among fly strains collected from human corpses. Notably, the control strain, which has been maintained for a long time in the laboratory environment, showed a relatively lower capability of debridement than any of the corpse-derived strains examined. Adaptation of the control strain to artificial breeding conditions through the mechanism of phenotypic plasticity may be considered^46,47^. It is also possible that fly larvae of the corpse-derived strains, which have ancestors that have recently experienced feeding on human tissues, may prefer necrotic human carrion resulting in ingesting a larger amount of these tissues. Variations in feeding amount and growth rate among the flies of even the same species suggested the importance of evaluating fly strains before and after their application for MDT.

Larvae of corpse-derived *L. sericata* strain (#28) ingested 40% more human tissue, grew larger, and reached the third instar and pupal stage almost 1 day earlier than the conventional medical maggots. Only first and early second instar larvae are applied to wounds or ulcers in clinical practice, so larvae ingesting a larger amount of tissue at the early stage may be beneficial for MDT. The enlarged cell size observed in the larval fat body and adult wing of the corpse-derived strain may explain its increased appetite and accelerated growth, although it remains unclear whether the cell enlargement is a cause or a consequence of its higher food intake ability. In MDT treatment, the maggots are usually replaced every 48 h and repeated for one to three courses, depending on the size of the wound surface. Application of the “gluttonous” maggots identified in this study to MDT may reduce the number of repeated courses, thus suggesting that the burden on the patient can be reduced. Another noteworthy feature observed in the corpse-derived strain was a remarkable effect of larval ES on the proliferation of HFF cells compared to the control strain. The effects of larval ES on the proliferation and migration of human fibroblasts have been previously proposed^16,18^. The larval ES may further promote granulation tissue formation by stimulating the proliferation of fibroblasts in human wounds. However, details of larval ES function, such as which compounds in larval ES exert wound-healing activity^48^, remain unknown and need further investigation.

A set of candidate genes possibly related to its higher abilities of larval feeding and stimulating cell proliferation was identified by analyzing DEGs in the corpse-derived strain. Notably, a *tribbles*-like gene was annotated as being more highly expressed in the corpse-derived strain. *Drosophila* Tribbles was first identified as pseudokinase playing roles in regulating cell proliferation and migration^49^. Contrary to the previous findings that *Drosophila* Tribbles regulates insulin signaling negatively and its overexpression led to decreased larval size and delayed maturation^50^, *L. sericata* Tribbles may function to enhance cell growth signaling at least in the larval stages. Indeed, complicated roles of Tribbles, occasionally transforming from a tumor suppressor gene into an oncogene, have been reported in several species, including humans and mice^51^. The germ-line transformation method for *L. sericata* has been established; a transgenic fly expressing human platelet-derived growth factor-BB was reported^52,53^. Genome editing in *L. cuprina*, a close relative of *L. sericata*, using the CRISPR/Cas9 system has also been reported, and its efficacy was demonstrated by disrupting the *yellow* gene^54^. Gene manipulation targeting these genes in *L. sericata* may provide detailed information about the cues responsible for eliciting the ingestion of more tissue and faster growth. Moreover, it may enable the establishment of manipulated high-performance fly strains appropriate for improved MDT to treat wounds and ulcers more effectively.

In this study, we established novel carrion blowfly strains, and a detailed analysis of the maggots’ feeding rate, wound healing effect, and gene expression suggested that they have the potential to be used for more effective MDT. All the results reported here are from in vitro experiments, and further in vivo analysis of therapeutic effects is expected for use in MDT on actual clinical applications.

## Conclusion

The present study showed that a newly established feeding assay using human tissue may be preferred to evaluate important parameters in medical maggots, focusing mainly on the amount of food ingested by carrion fly larvae. It was also revealed that the corpse-derived strains had higher feeding ability, and one of these strains was capable of stimulating human cell proliferation via its larval ES more efficiently than the standard medical strain. Validating the feeding ability of medical maggots by employing human tissues and introducing new fly strains collected from human corpses may offer a promising strategy to secure and improve the quality of fly larvae, which may affect the outcome of MDT.

## Supplementary Information

Supplementary Information.

## Data Availability

RNA-Seq data have been deposited in DRA (http://trace.ddbj.nig.ac.jp/dra) under accession numbers DRA013344. All other data generated or analyzed during this study are included in this published article (and its Supplementary Information files) or are available from the corresponding author on reasonable request.

## References

[1] World Health Organization. Global report on diabetes. https://www.who.int/publications/i/item/9789241565257 (2016).

[2] Bhurosy T, Jeewon R (2014). Overweight and obesity epidemic in developing countries: A problem with diet, physical activity, or socioeconomic status?. ScientificWorldJournal..

[3] Misra A, Ramchandran A, Jayawardena R, Shrivastava U, Snehalatha C (2014). Diabetes in South Asians. Diabet. Med..

[4] Boulton AJ, Vileikyte L, Ragnarson-Tennvall G, Apelqvist J (2005). The global burden of diabetic foot disease. Lancet.

[5] García-Morales E, Lázaro-Martínez JL, Martínez-Hernández D, Aragón-Sánchez J, Beneit-Montesinos JV, González-Jurado MA (2011). Impact of diabetic foot related complications on the Health Related Quality of Life (HRQol) of patients—a regional study in Spain. Int. J. Low Extrem Wounds..

[6] Peters EJ, Childs MR, Wunderlich RP, Harkless LB, Armstrong DG, Lavery LA (2001). Functional status of persons with diabetes-related lower-extremity amputations. Diabetes Care.

[7] Hambleton IR, Jonnalagadda R, Davis CR, Fraser HS, Chaturvedi N, Hennis AJ (2009). All-cause mortality after diabetes-related amputation in Barbados: A prospective case-control study. Diabetes Care.

[8] Gurney JK, Stanley J, York S, Rosenbaum D, Sarfati D (2018). Risk of lower limb amputation in a national prevalent cohort of patients with diabetes. Diabetologia.

[9] Jupiter DC, Thorud JC, Buckley CJ, Shibuya N (2016). The impact of foot ulceration and amputation on mortality in diabetic patients. I: From ulceration to death, a systematic review. Int. Wound J..

[10] Thorud JC, Plemmons B, Buckley CJ, Shibuya N, Jupiter DC (2016). Mortality after nontraumatic major amputation among patients with diabetes and peripheral vascular disease: A systematic review. J. Foot Ankle Surg..

[11] Paré, A., Johnson, T. & Spiegel, A. The works of that famous chirurgeon Ambrose Parey (1678) 10.5962/bhl.title.153571.

[12] Paul AG, Ahmad NW, Lee HL, Ariff AM, Saranum M, Naicker AS, Osman Z (2009). Maggot debridement therapy with *Lucilia*
*cuprina*: A comparison with conventional debridement in diabetic foot ulcers. Int. Wound J..

[13] Andersen AS, Sandvang D, Schnorr KM, Kruse T, Neve S, Joergensen B, Karlsmark T, Krogfelt KA (2010). A novel approach to the antimicrobial activity of maggot debridement therapy. J. Antimicrob. Chemother..

[14] Beasley WD, Hirst G (2004). Making a meal of MRSA-the role of biosurgery in hospital-acquired infection. J. Hosp. Infect..

[15] Robinson W, Norwood VH (1933). The rôle of surgical maggots in the disinfection of osteomyelitis and other infected wounds. J. Bone Joint Surg..

[16] Prete PE (1997). Growth effects of *Phaenicia sericata* larval extracts on fibroblasts: Mechanism for wound healing by maggot therapy. Life Sci..

[17] Zhang Z, Wang S, Diao Y, Zhang J, Lv D (2010). Fatty acid extracts from *Lucilia sericata* larvae promote murine cutaneous wound healing by angiogenic activity. Lipids Health Dis..

[18] Horobin AJ, Shakesheff KM, Pritchard DI (2005). Maggots and wound healing: An investigation of the effects of secretions from *Lucilia sericata* larvae upon the migration of human dermal fibroblasts over a fibronectin-coated surface. Wound Repair Regen..

[19] Dallavecchia DL, da Silva Filho RG, Aguiar VM (2014). Sterilization of *Chrysomya putoria* (Insecta: Diptera: Calliphoridae) eggs for use in biotherapy. J. Insect Sci..

[20] de Masiero FS, Nassu MP, Soares MP, Thyssen PJ (2015). Histological patterns in healing chronic wounds using *Cochliomyia macellaria* (Diptera: Calliphoridae) larvae and other therapeutic measures. Parasitol. Res..

[21] Díaz-Roa A, Gaona MA, Segura NA, Ramírez-Hernández A, Cortés-Vecino JA, Patarroyo MA, Bello F (2016). Evaluating *Sarconesiopsis magellanica* blowfly-derived larval therapy and comparing it to *Lucilia sericata*-derived therapy in an animal model. Acta Trop..

[22] Sun X, Jiang K, Chen J, Wu L, Lu H, Wang A, Wang J (2014). A systematic review of maggot debridement therapy for chronically infected wounds and ulcers. Int. J. Infect. Dis..

[23] Food and Drug Administration. 510(k) Summary. K072438. (2007) https://www.accessdata.fda.gov/cdrh_docs/pdf7/K072438.

[24] Sherman RA, Mumcuoglu KY, Grassberger M, Tantawi TI, Grassberger M, Sherman R, Gileva O, Kim C, Mumcuoglu K (2013). Maggot therapy. Biotherapy—History, Principles and Practice: A Practical Guide to the Diagnosis and Treatment of Disease using Living Organisms.

[25] Okada T, Mitsui H (2015). A way of using maggot therapy at outpatient clinic. J. Jpn. Plast. Surg..

[26] Sherman RA (2009). Maggot therapy takes us back to the future of wound care: New and improved maggot therapy for the 21st century. J. Diabetes Sci. Technol..

[27] Blake FA, Abromeit N, Bubenheim M, Li L, Schmelzle R (2007). The biosurgical wound debridement: Experimental investigation of efficiency and practicability. Wound Repair Regen..

[28] Čičková H, Kozánek M, Takáč P (2015). Growth and survival of blowfly *Lucilia sericata* larvae under simulated wound conditions: Implications for maggot debridement therapy. Med. Vet. Entomol..

[29] Wilson MR, Nigam Y, Knight J, Pritchard DI (2019). What is the optimal treatment time for larval therapy? A study on incubation time and tissue debridement by bagged maggots of the greenbottle fly, *Lucilia*
*sericata*. Int. Wound J..

[30] Cole J (2017). Assessing the calorific significance of episodes of human cannibalism in the Palaeolithic. Sci. Rep..

[31] Williams KA, Villet MH (2014). Morphological identification of *Lucilia sericata*, *Lucilia cuprina* and their hybrids (Diptera, Calliphoridae). Zookeys..

[32] Williams KA, Lamb J, Villet MH (2016). Phylogenetic radiation of the greenbottle flies (Diptera, Calliphoridae, Luciliinae). Zookeys..

[33] Saigusa K, Takamiya M, Aoki Y (2005). Species identification of the forensically important flies in Iwate prefecture, Japan based on mitochondrial cytochrome oxidase gene subunit I (COI) sequences. Leg Med..

[34] Williams, K. A. & Villet, M. H. Ancient and modern hybridization between *Lucilia sericata* and *L. cuprina* (Diptera: Calliphoridae). *Eur. J. Entomol.* **110** (2013).

[35] Martin M (2011). Cutadapt removes adapter sequences from high-throughput sequencing reads. Embnet. J..

[36] Bolger AM, Lohse M, Usadel B (2014). Trimmomatic: A flexible trimmer for Illumina sequence data. Bioinformatics.

[37] Grabherr MG, Haas BJ, Yassour M, Levin JZ, Thompson DA, Amit I, Adiconis X, Fan L, Raychowdhury R, Zeng Q, Chen Z, Mauceli E, Hacohen N, Gnirke A, Rhind N, di Palma F, Birren BW, Nusbaum C, Lindblad-Toh K, Friedman N, Regev A (2011). Full-length transcriptome assembly from RNA-Seq data without a reference genome. Nat. Biotechnol..

[38] Li B, Dewey CN (2011). RSEM: Accurate transcript quantification from RNA-Seq data with or without a reference genome. BMC Bioinform..

[39] Robinson MD, McCarthy DJ, Smyth GK (2010). edgeR: A Bioconductor package for differential expression analysis of digital gene expression data. Bioinformatics.

[40] Midgley JM, Villet MH (2009). Effect of the killing method on post-mortem change in length of larvae of *Thanatophilus micans* (Fabricius 1794) (Coleoptera: Silphidae) stored in 70% ethanol. Int. J. Legal Med..

[41] Warren JA, Ratnasekera TDP, Campbell DA, Anderson GS (2018). Hyperspectral measurements of immature *Lucilia sericata* (Meigen) (Diptera: Calliphoridae) raised on different food substrates. PLoS ONE.

[42] Clark K, Evans L, Wall R (2006). Growth rates of the blowfly, *Lucilia sericata*, on different body tissues. Forensic Sci. Int..

[43] Wei JCJ, Edwards GA, Martin DJ, Huang H, Crichton ML, Kendall MAF (2017). Allometric scaling of skin thickness, elasticity, viscoelasticity to mass for micro-medical device translation: From mice, rats, rabbits, pigs to humans. Sci. Rep..

[44] MacInnis AE, Higley LG (2020). Competition among three forensically important blow fly species (Diptera: Calliphoridae): *Phormia*
*regina*, *Lucilia*
*sericata*, and *Chrysomya*
*rufifacies*. Environ. Entomol..

[45] Gallagher MB, Sandhu S, Kimsey R (2010). Variation in developmental time for geographically distinct populations of the common green bottle fly, *Lucilia*
*sericata* (Meigen). J. Forensic Sci..

[46] Darwin C (1868). The Variation of Animals and Plants under Domestication.

[47] Lande R (2009). Adaptation to an extraordinary environment by evolution of phenotypic plasticity and genetic assimilation. J. Evol. Biol..

[48] Honda K, Okamoto K, Mochida Y, Ishioka K, Oka M, Maesato K, Ikee R, Moriya H, Hidaka S, Ohtake T, Doi K, Fujita T, Kobayashi S, Noiri E (2011). A novel mechanism in maggot debridement therapy: Protease in excretion/secretion promotes hepatocyte growth factor production. Am. J. Physiol. Cell Physiol..

[49] Mata J, Curado S, Ephrussi A, Rørth P (2000). Tribbles coordinates mitosis and morphogenesis in Drosophila by regulating string/CDC25 proteolysis. Cell.

[50] Das R, Sebo Z, Pence L, Dobens LL (2014). Drosophila tribbles antagonizes insulin signaling-mediated growth and metabolism via interactions with Akt kinase. PLoS ONE.

[51] Dobens LL, Nauman C, Fischer Z, Yao X (2021). Control of cell growth and proliferation by the tribbles pseudokinase: Lessons from Drosophila. Cancers.

[52] Concha C, Belikoff EJ, Carey BL, Li F, Schiemann AH, Scott MJ (2011). Efficient germ-line transformation of the economically important pest species *Lucilia*
*cuprina* and *Lucilia*
*sericata* (Diptera, Calliphoridae). Insect Biochem. Mol. Biol..

[53] Linger RJ, Belikoff EJ, Yan Y, Li F, Wantuch HA, Fitzsimons HL, Scott MJ (2016). Towards next generation maggot debridement therapy: Transgenic *Lucilia*
*sericata* larvae that produce and secrete a human growth factor. BMC Biotechnol..

[54] Paulo DF, Williamson ME, Arp AP, Li F, Sagel A, Skoda SR, Sanchez-Gallego J, Vasquez M, Quintero G, Pérez de León AA, Belikoff EJ, Azeredo-Espin AML, McMillan WO, Concha C, Scott MJ (2019). Specific gene disruption in the major livestock pests *Cochliomyia*
*hominivorax* and *Lucilia*
*cuprina* using CRISPR/Cas9. G3.

